# Hardware Validation of the Advanced Plant Habitat on ISS: Canopy Photosynthesis in Reduced Gravity

**DOI:** 10.3389/fpls.2020.00673

**Published:** 2020-06-18

**Authors:** Oscar Monje, Jeffrey T. Richards, John A. Carver, Dinah I. Dimapilis, Howard G. Levine, Nicole F. Dufour, Bryan G. Onate

**Affiliations:** ^1^AECOM Management Services Inc., LASSO, Kennedy Space Center, Merritt Island, FL, United States; ^2^TOSC, Jacobs Technology, Kennedy Space Center, Merritt Island, FL, United States; ^3^NASA, UB-A, Kennedy Space Center, Merritt Island, FL, United States

**Keywords:** Apogee Wheat, *Arabidopsis*, canopy photosynthesis, controlled environment agriculture, elevated CO_2_, ISS spaceflight environment, NASA APH, microgravity

## Abstract

The Advanced Plant Habitat (APH) is the largest research plant growth facility deployed on the International Space Station (ISS). APH is a fully enclosed, closed-loop plant life support system with an environmentally controlled growth chamber designed for conducting both fundamental and applied plant research during experiments extending as long as 135 days. APH was delivered to the ISS in parts aboard two commercial resupply missions: OA-7 in April 2017 and SpaceX-11 in June 2017, and was assembled and installed in the Japanese Experiment Module Kibo in November 2018. We report here on a 7-week-long hardware validation test that utilized a root module planted with both *Arabidopsis* (cv. Col 0) and wheat (cv. Apogee) plants. The validation test examined the APH’s ability to control light intensity, spectral quality, humidity, CO_2_ concentration, photoperiod, temperature, and root zone moisture using commanding from ground facilities at the Kennedy Space Center (KSC). The test also demonstrated the execution of programmed experiment profiles that scheduled: (1) changes in environmental combinations (e.g., a daily photoperiod at constant relative humidity), (2) predetermined photographic events using the three APH cameras [overhead, sideview, and sideview near-infrared (NIR)], and (3) execution of experimental sequences during the life cycle of a crop (e.g., measure photosynthetic CO_2_ drawdown experiments). *Arabidopsis* and wheat were grown in microgravity to demonstrate crew procedures, planting protocols and watering schemes within APH. The ability of APH to contain plant debris was assessed during the harvest of mature *Arabidopsis* plants. Wheat provided a large evaporative load that tested root zone moisture control and the recovery of transpired water by condensation. The wheat canopy was also used to validate the ability of APH to measure gas exchange of plants from non-invasive gas exchange measurements (i.e., canopy photosynthesis and respiration). These features were evaluated by executing experiment profiles that utilized the CO_2_ drawdown technique to measure daily rates of canopy photosynthesis and dark-period CO_2_ increase for respiration. This hardware validation test confirmed that APH can measure fundamental plant responses to spaceflight conditions.

## Introduction

**FIGURE 1:**
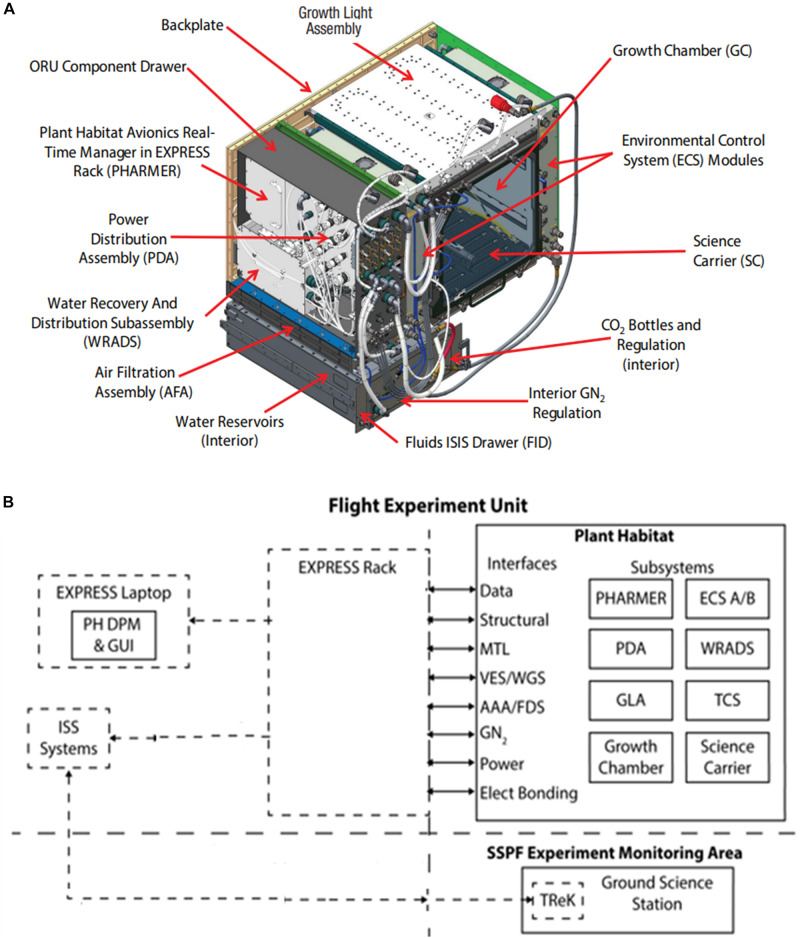
APH Facility: **(A)** Modular APH subsystems. **(B)** Overall APH system architecture. Abbreviations are listed in Table 1.

The Advanced Plant Habitat (APH) facility was designed and built by NASA and Orbital Technologies Corporation (ORBITEC, now Sierra Nevada Corp., Madison, WI, United States) to conduct both fundamental and applied plant research in reduced gravity (Figure 1). Located in the Kibo module on the ISS, the APH was designed for a 10-year mission for collecting physiological data of plant responses to the spaceflight environment. This data will improve our understanding of how terrestrial biology responds to reduced gravity which is useful for enabling the manned exploration of space (National Research Council, 2011; Wheeler, 2017).

This paper describes the results of a hardware validation technology demonstration of the APH facility after its assembly on ISS. Technology demonstrations are simpler than typical flight experiments conducting peer-reviewed science designed to discern differences in plant responses caused by the lack of gravity (Stutte et al., 2015). As such, extensive science and experiment verification tests prior to flight, and a corresponding ground experiment mimicking the culture and environmental conditions experienced on ISS were not conducted.

The goal of this hardware validation test was to demonstrate that the APH facility was fully operational and capable of conducting fundamental plant research in microgravity onboard the ISS. The functionality of APH subsystems was verified, as well as the ability of APH to grow two plant species in microgravity during a 7-week period. Minimal ground testing was conducted in an APH engineering development unit (i.e., a similar but non-flight qualified version of the APH) to ensure wheat and *Arabidopsis* plants would germinate using the watering protocols recommended by the APH design team. The operation of the APH facility was verified by executing experiment profiles that controlled: (1) the environmental conditions during growth of each plant species, (2) chamber CO_2_ to obtain daily gas exchange data from a plant canopy, and (3) chamber CO_2_ and light intensity to conduct pre-programmed CO_2_ drawdown experiments.

### Spaceflight Plant Research

The primary goal of several decades of spaceflight plant research has been to determine if plant growth, development, and reproduction in microgravity is similar to that on Earth (Stutte et al., 2015; Zabel et al., 2016). The goal of this research was to determine if future plant-based Bioregenerative Life Support Systems (BLSS) growing crops for human colonies on the Moon or Mars (Averner et al., 1984; Wheeler, 2010, 2017) can be sized using 1 g data (Monje et al., 2005). In addition, NASA has recently identified the need for new technologies in space crop production and food safety for supplementing the space diet with fresh leafy green crops in near term ISS, cislunar, and lunar missions (Massa et al., 2015; Anderson et al., 2017).

Spaceflight plant research is conducted in plant chambers designed to operate in reduced gravity. Previous spaceflight experiments found that indirect effects of microgravity reduce plant growth in space (Musgrave et al., 1997; Monje et al., 2003). These indirect effects include: (1) altered behavior of liquids and gases affecting fluid behavior phenomena, (2) capillary-driven moisture redistribution resulting in poor root-zone aeration, and (3) the absence of buoyancy-driven convection causing poor mass and heat transfer to leaves and plant organs (Musgrave et al., 1997; Musgrave, 2002; Monje et al., 2003; Heinse et al., 2007; Kitaya et al., 2010; Stutte et al., 2015). Other considerations include the limited availability of resources in spacecraft (i.e., mass, power and volume) for plant chambers (Zabel et al., 2016). As a result, plant growth systems for conducting spaceflight experiments [e.g., Advanced Astroculture (ADVASC) and the Biomass Production System (BPS)] were designed to mitigate these secondary effects of the spaceflight environment using forced convection and actively watered root modules that provide well-aerated root zones (Stutte et al., 2015; Zabel et al., 2016). However, these systems were limited by small growth areas available for crop production. Larger plant growth systems are required to overcome the remaining challenges in spaceflight plant research. These challenges are to develop and demonstrate the performance of substrate-free, gravity-independent, water delivery systems to safely grow salad crops in reduced gravity environments for supplementing crew diets (Massa et al., 2015; Anderson et al., 2017; Monje et al., 2019; Khodadad et al., 2020).

### NASA Facilities Enable Future Exploration

NASA recently developed two new plant research facilities, Veggie and the APH, for conducting spaceflight plant research on ISS as recommended by the NRC Decadal Survey Study “Recapturing a Future for Space Exploration: Life and Physical Sciences Research for a New Era” (National Research Council, 2011). These facilities have larger plant growth areas (∼0.2 m^2^) and volumes than previous platforms, and are designed for studying crop production, plant-to-plant interactions, and human-plant-microbial ecosystems using large plants in space (Massa et al., 2016; Khodadad et al., 2020). Veggie uses LED lights, a passive watering system and minimal environmental control consisting of a fan to circulate ISS air through the plant growth volume. Recently, the Veggie was used to grow nutritious lettuce crops that are safe to eat on ISS (Khodadad et al., 2020). In contrast, the APH facility is a research, plant growth chamber that can grow plants under complete environmental control (i.e., spectral quality, light intensity, temperature, relative humidity, CO_2_ and ethylene concentration) for life cycles as long as 135 days (Morrow et al., 2016). It incorporates a root module watering design that is similar to those developed for ADVASC and BPS: a substrate-based water delivery system that actively controls matric potential of the root zone (Morrow and Crabb, 2000; Link et al., 2003; Morrow et al., 2004).

The APH has the ability to collect non-destructive data sets (i.e., images and gas exchange rates) for measuring plant growth during spaceflight, and significantly expands the lighting capabilities (e.g., higher light intensities and increased spectral combinations) available for plant research on ISS. The APH measures CO_2_ and water vapor fluxes using non-destructive gas exchange techniques demonstrated on the BPS, a predecessor to the APH with an identical CO_2_ control architecture (Monje et al., 2005). During the ISS Expedition 4 in 2002, the BPS was used to measure the photosynthetic canopy quantum yield (CQY), the conversion of absorbed radiation into gross CO_2_ fixation, of wheat plant stands in microgravity using the CO_2_ drawdown technique (Morrow et al., 2004, 2016; Monje et al., 2005; Stutte et al., 2005). In that study, the canopy photosynthetic rates and CQY of 21-day-old wheat in microgravity did not differ from 1g controls at moderate light intensities (Stutte et al., 2005). The APH was designed to also measure canopy photosynthetic rates and CQY during spaceflight, however, at higher light intensities than previously available in the BPS. The APH provides lighting with a wide range in spectral quality and a maximum photosynthetic photon flux density (PPFD) of 1,000 μmol m^–2^ s^–1^, which is nearly three times higher than was possible in the BPS.

Future plant research conducted in Veggie and APH will enable exploration by improving our understanding of how plants, and their associated microbiomes in leaves and roots, grow in the spaceflight environment. This knowledge is useful for developing suitable countermeasures to mitigate potential problems of crop production, water recycling, and atmosphere revitalization needed for supporting sustainable and long-term human colonies in space (Wheeler, 2010, 2017).

### APH Facility

The APH facility is a platform that permits the collection of physiological and environmental data to test specific hypotheses on how plant development, gene expression, photosynthesis and respiration, seed formation, plant/microbe interactions, or growth rate may respond to primary effects of microgravity or to secondary effects of the spaceflight environment. The APH is a controlled environment chamber (0.2 m^2^ by 0.4 m tall) that is teleoperated from the ground, permitting minimal crew involvement to conduct science (Figure 1A). Crew operations are limited to adding water to the APH reservoirs, collecting biological samples, harvesting plants, and conducting periodic system maintenance. Biological samples from shoots and roots grown in APH (such as leaf disks, periodic harvest of plants, etc.) can be returned frozen, or in Kennedy Fixation Tubes (KFTs) containing fixatives (e.g., RNAlater), for post-flight analysis on the ground.

The growth chamber controls temperature (T; 18–30°C), relative humidity (RH; 50–90%), CO_2_ concentration (400–5,000 μmol mol^–1^), wind speed (0.3–1.5 m s^–1^), spectral quality, and provides the highest light level (up to 1,000 μmol m^–2^ s^–1^) of any spaceflight chamber to date (Massa et al., 2016; Zabel et al., 2016). In its baseline configuration, the plants germinate and grow in the APH Science Carrier (SC), a four-quadrant rooting module, using fertilized substrate-based media. Water for filling the reservoirs and wetting the rooting media are initially supplied from ISS potable water, while the majority of the water for sustaining plant growth and operations is provided by condensed humidity that is recycled back to the reservoir. Soil (media) water content is controlled in each rooting quadrant using an active moisture control system.

Currently, the baseline configuration of the SC is a single-use 0.2 m^2^ module that uses a porous granular substrate (1–2 mm arcillite) as rooting media. The media is watered using a manifold of porous ceramic tubes, and moisture content is actively controlled with negative pressure (i.e., suction) to provide optimum root zone water and O_2_ in microgravity (Monje et al., 2005; Stutte et al., 2005; Morrow et al., 2016). The amount of consumable media (∼4 kg per experiment) required by the current APH SC configuration makes this growth system unsustainable for future food production missions beyond low Earth orbit (Monje et al., 2019), but it is adequate for conducting space biology and life-cycle crop production experiments. In space biology experiments, used root modules are often discarded after the plants are harvested because sample return space and down mass from the ISS is limited, however, the APH facility is versatile and can accommodate alternative SC designs for testing future sustainable crop production systems.

### APH Architecture

**FIGURE 2:**
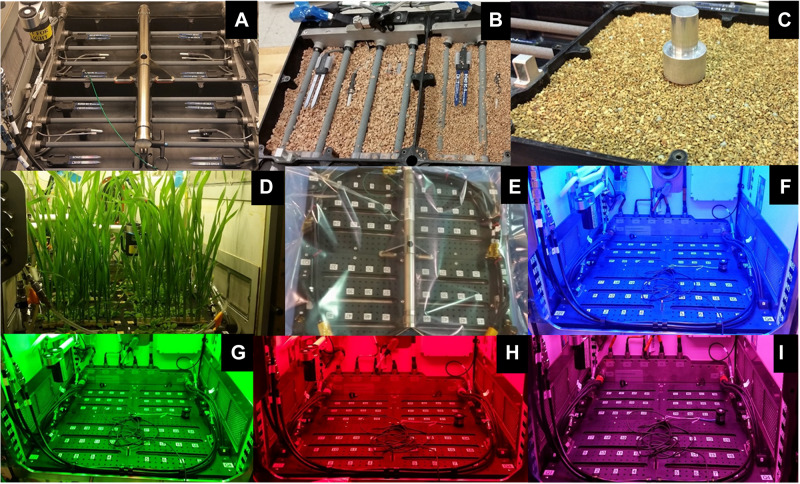
APH Growth chamber subsystems: Science Carrier [SC; **(A–E)**] and Growth Light Assembly [GLA; **(F–I)**]. The SC is packed with 1–2 mm arcillite **(A–C)**. The planting, germination and cultivation procedures were demonstrated in APH during ground studies **(D)**. The Flight SC was shipped wrapped in a Tedlar bag **(E)**. The GLA illuminates plants with LED lighting of different wavelengths: blue 455 nm **(F)**, green 530 nm **(G)**, red 630 nm **(H)**, far-red 735 nm, and white 4100 K. The GLA can also provide mixed light recipes: red, blue, and white **(I)**.

The APH facility (Figure 1A) consists of the PHARMER (Plant Habitat Avionics Realtime Manager in EXPRESS Rack see Table 1 for abbreviations) that commands several APH subsystems, including the Growth Chamber (GC) and the Growth Light Assembly (GLA). The GC controls ambient CO_2_ concentration, removes ethylene, and houses the SC root module (Figures 2A–C). The PHARMER subsystem interfaces with other APH subsystems to monitor and control the internal APH environment and fluid levels along with APH thermal and power paths.

**TABLE 1:** List of abbreviations.

Symbol	Definition
AAA	Avionics air assembly
ADVSC	Advance Astroculture
APH	Advance Plant Habitat
BLSS	Bioregenerative life support systems
BPS	Biomass Production System
CQY	Canopy quantum yield
CWF	Cool white fluorescent
DAP	Days after planting
DPM	Data processing module
EMA	Experiment Monitoring Area
ECS	Environmental control subsystems
FDS	Fire detection system
GC	Growth chamber
GLA	Growth light assembly
GUI	Graphical user interface
HPS	High pressure sodium
ISS	International Space Station
KFT	Kennedy fixation tubes
KSC	Kennedy Space Center
MTL	Moderate temperature loop
NIR	Near-infrared
ORU	Orbital replacement unit
PH-01	Plant Habitat 1 experiment
PHARMER	Plant habitat avionics realtime manager in EXPRESS rack
PDA	Power distribution assembly
P_*net*_	Canopy net photosynthetic rate
PPFD	Photosynthetic photon flux density
R_*dark*_	Canopy dark respiration rate
SC	Science carrier
TCS	Thermal control subsystem
TReK	Telescience resource kit software
VES	Vacuum exhaust system
WGS	Waste gas system
WRADS	Water recovery and distribution subsystem

The APH has two Environmental Control Subsystems (ECS) that physically interface to the EXPRESS Rack Gaseous Nitrogen (GN_2_) system, Avionics Air Assembly (AAA), and Moderate Temperature Loop (MTL) (Figure 1B). The ECS units control GC air circulation, filtration, and provide temperature and humidity conditioning. The Water Recovery and Distribution Subsystem (WRADS) operates in conjunction with the APH ECS to provide water to the plant growth chamber. The APH facility has two water reservoirs (Distribution and Recovery) with a combined volume of 3 L. Plants growing in the GC transpire water supplied to the root zone from the 2 L Distribution reservoir. Transpired water is condensed and stored in the 1 L Recovery reservoir. The ECS replenishes the Recovery reservoir with condensate water and the WRADS dispenses water stored in the Distribution reservoir to each quadrant of the SC. The APH Power Distribution Assembly (PDA) is powered via the EXPRESS Rack power interface and provides the appropriate power levels to APH components: PHARMER, GC, GLA, SC, ECS, WRADS, and the Thermal Control Subsystem (TCS).

The PHARMER also provides the communication path for command uplink to APH and telemetry downlink from APH via the EXPRESS Rack. Commanding of APH from the ground (i.e., teleoperation) was conducted at the Experiment Monitoring Area (EMA) at KSC.

## Hardware Validation

A timeline of the APH hardware validation events is summarized in Table 2. The APH Flight Unit 1 was shipped in parts to the ISS on two spacecraft and assembled in space by the crew. After APH was powered up, a series of functional tests were conducted over 5 days (November 27 to December 01, 2017) to validate that the hardware was operational. The PHARMER was initiated, then commanding, telemetry, and data retrieval from PHARMER was verified, and each APH subsystem was powered up sequentially. The water reservoirs were filled by the crew with ISS potable water and the environmental control functions (temperature, humidity, CO_2_ concentration, spectral quality, and light intensity), as well as, camera control functions of APH, were validated via commanding from the ground at KSC. At the end of the functional test, an acoustic test was performed and the APH was then configured for conducting a plant experiment.

**TABLE 2:** Summary of events during hardware validation of APH on ISS.

Event/Test	Spacecraft/Module	Date	Astronaut
Transported to ISS	Cygnus OA-7	April 18, 2017	
	SpaceX 11	June 02, 2017	
Assembly	Kibo	October 27, 2017	Joe Acaba
1st Power up	5 Day functional	November 27 to December 01, 2017	Joe Acaba
Acoustic test	Kibo	December 08, 2017	Alexander Misurkin
1st Plant test	*Arabidopsis*	January 22, 2018 to March 06, 2018	Norishige Kanai
	Wheat	February 08, 2018 to March 12, 2018	Norishige Kanai

### First Power-Up

After the APH facility was powered up, the crew filled the Recovery and Distribution reservoirs manually with potable galley water. Thereafter, the remaining activation steps were executed by an operator via commanding from the ground in the EMA at KSC using software and communications channeled via the Marshall Spaceflight Center. The environmental control systems (ECS-A and ECS-B) that control chamber temperature and humidity were primed and adjusted. Once stable temperature and humidity controls were established, it was verified that the ECS modules could control chamber temperature and humidity at the following setpoints: 23°C/70% RH, 18°C/50% RH, 18°C/90% RH, 30°C/90% RH, and 30°C/50% RH.

The GC subsystem was powered to enable the chamber pressure, O_2_, and CO_2_ sensors, as well as, initiate CO_2_ and ethylene control. The APH GLA can generate a wide variety of spectral quality recipes using five LED banks: blue (0–400 μmol m^–2^ s^–1^ at 450 nm), green (0–100 μmol m^–2^ s^–1^ at 525 nm), red (0–600 μmol m^–2^ s^–1^ at 630 nm), white (0–600 μmol m^–2^ s^–1^ at 400–740 nm), and near-infrared (0–50 μmol m^–2^ s^–1^ at 735 nm). The setpoints for each separate blue, green, white, red, and near-infrared LEDs of the GLA were adjusted one at a time and photographs of the illuminated growth chamber were taken to confirm their operation (Figures 2F–H).

The APH operates in several modes: Standby, Manual, and Experiment Profile. To test the Experiment Profile mode, the APH was configured to a desired experimental profile that adjusts daily settings of light intensity, spectral quality (Figure 2I), photoperiod, thermoperiod, relative humidity (RH), CO_2_ concentration, chamber air speed, ethylene removal, and root zone matric potential. Changes in the timing of these parameters were observed, recorded, and verified using the APH Command log.

### First Plant Test Overview

The main purpose of the first on-orbit plant growth test was to validate that the APH could grow large plants in the spaceflight environment. However, plant growth during spaceflight could not be compared to 1g controls because an identical ground study was not conducted during the validation test. Furthermore, there was no crew time allotted for thinning plants, and there were no provisions to bring back harvested plant samples for analysis on the ground. Instead, non-destructive and non-invasive gas exchange measurements (i.e., photosynthesis and respiration) of an *Arabidopsis*/wheat canopy were recorded daily. Thus, healthy plant growth was ascertained by comparing images and gas exchange data collected during the life cycle of the plants with known values reported in the literature. The photosynthetic responses of an *Arabidopsis*/wheat canopy to light and CO_2_ concentration were measured in microgravity, and compared to literature values from previous studies with wheat conducted at 1g and during spaceflight (Wheeler, 1996; Monje and Bugbee, 1998; Stutte et al., 2005).

A SC root module, composed of 4 quadrants packed with 1–2 mm arcillite (Figures 2A–C), was pre-planted with two quadrants of *Arabidopsis* (WT) cv Col-0 and two quadrants of wheat cv Apogee. The SC planting and watering protocols used were previously tested on the ground in an APH engineering development unit (Figure 2D). For the 1st plant test of APH in microgravity, the pre-planted SC was wrapped in a Tedlar bag, packed in soft foam shipping units, and launched to ISS (Figure 2E) on Cygnus OA-7.

During the first plant test, the SC was removed from the Tedlar stowage bag and installed in the APH by the crew. The APH was then teleoperated from the ground to water the plants and collect plant growth data without crew involvement. Experiment profiles, scripts that are executed daily by the PHARMER, were uploaded and implemented to capture a daily record of images, environmental and plant performance data. The experiment profiles were also used for conducting pre-programmed CO_2_ drawdown experiments to collect non-destructive gas exchange data. The data collected included GC and SC environmental parameters, daily overhead and sideview images, and daily measurements of photosynthesis and respiration. In addition, the experiment profiles controlled the photoperiod in the GC and the media moisture content in each SC quadrant. The drawdown experiment generated plant response curves to altered CO_2_ concentrations and light intensities. Thus, a major portion of the science capabilities of APH were verified during the first plant test.

## APH Components

### Environmental Control Systems (ECS)

Two ECS modules, mounted on each side of the growth chamber, control chamber temperature, humidity, and air flow (Figure 1A). Each ECS first condenses/humidifies chamber air using moist porous ceramic cups under suction, and then heats the air to the desired setpoint temperature. Each unit is independently monitored and controlled, and has the capability to independently control temperature in the growth chamber from 18 to 30°C (±1°C), and relative humidity from 50 to 90% (±5%). Preparing the two ECS units for operation required priming with water (∼0.8 L) up to the porous ceramic cups in order for RH and temperature control to operate efficiently. Pressure control setpoints for the porous cups were gradually set to start recovery (or addition) of water to adjust the relative humidity of the airflow through each unit.

The ECS modules mix the air in the growth chamber by forced convection using fans that remove air from the top of the growth chamber above the plants and return it into the chamber at plant level from opposing sides of the chamber. Fan speeds are controllable from 0.3 to 1.5 m s^–1^ at 0.1 m s^–1^ increments. Replaceable HEPA filters prevent particulates and plant debris from contaminating the internal components of the ECS modules during nominal operating conditions. A goal of the first plant experiment was to demonstrate how the ECS filters would capture plant debris, especially that generated during the growth and harvest of mature *Arabidopsis* plants.

### Science Carrier (SC) – Preparation and Planting

The APH WRADS independently controls moisture content of the four quadrants in the SC (Morrow et al., 2016). The WRADS supplies water to each quadrant via a manifold that has four porous ceramic tubes (Refractron, Newark, NY, United States) embedded into the growth media. Each quadrant is first saturated and then drained to a desired moisture content. The WRADS measures the matric potential of the media in each quadrant using a pressure sensor, and root zone moisture setpoint is controlled by removing/adding water to the root module. Two additional capacitance-based moisture sensors (ECH_2_O EC-5, METER Group, United States) located vertically in each quadrant measure the corresponding volumetric moisture content of the media. One sensor is located above and the other is located below the porous tubes delivering water from the WRADS (Figure 2A). These sensors are not part of the moisture control loop but provide an independent method to gauge adequate watering of the media.

#### Media Preparation

The SC used a porous granular substrate media (arcillite; Turface Pro League Elite, Profile Products, LLC) to provide root anchoring, as well as storage and delivery of water, oxygen, and nutrients to plant roots. Arcillite was first sieved to a particle size of 1–2 mm and then washed with DI water to remove dust. Washing the arcillite was necessary for both crew safety and for reducing the potential for particulate clogging of the porous tubes in the SC quadrants. Sifted and washed arcillite was autoclaved in a covered tray for 30 min and dried in a forced air oven at 70°C for a minimum of 72 h until thoroughly dry.

Dried and sterile 1–2 mm arcillite was weighed (∼990 g per quadrant; equivalent to 1.6 L) into clean ziplock bags, and mixed gently with 7.5 g/L of Type 180, 18-6-8 Nutricote (Florikan ESA, Sarasota, FL, United States) time-release fertilizer pellets. A 1.6 L volume of media filled one of the four quadrants to the proper density and height in the SC (Figures 2A,B). The arcillite was carefully tamped down around the temperature and moisture sensors, as well as the porous watering tubes residing in each quadrant, to prevent movement of the clay particles during launch (Figure 2C). Once all four quadrants were filled with media, two quadrants were planted with *Arabidopsis* wild-type (WT) cv Col-0 seeds and two quadrants planted with wheat cv Apogee seeds. The planting and germination protocols for growing for *Arabidopsis* or wheat were different, so each system is described individually.

#### *Arabidopsis* Planting System

Extensive ground testing was conducted to determine the optimum planting and germination protocols for growing *Arabidopsis* on APH. The chosen protocol for germination of *Arabidopsis* employed a single layer of medical gauze. An individual medical gauze sheet (Covidien Curity, 4 in × 4 in, 4 ply; Medtronic, Minneapolis, MN, United States) was unfolded and placed on top of the tightly packed arcillite. The gauze was covered with washed and sterilized open-celled Roylan orthopedic foam (Performance Heath, Warrenville, IL, United States). The foam covered the majority of each quadrant except for precut strips covered with gauze where the seeds were planted. The foam, medical gauze, and arcillite were secured in place by a polycarbonate cover that contained three centered rows for planting and holes for aeration of the entire quadrant.

Supplementary ground testing of this planting and germination protocol was performed in collaboration with Washington State University (WSU) scientists to ensure the success of the first scientific study to be conducted on APH (PH-01): ‘An Integrated Omics Guided Approach to Lignification and Gravitational Responses: The Final Frontier’ (Lewis et al., 2020).

Wild Type (WT) *Arabidopsis* seeds cv Col-0 were surface sanitized with successive 70 and 95% ethanol washes, followed by overnight drying, prior to gluing onto the medical gauze. Individual seeds were first dipped onto a small drop of a sterile 1% guar gum solution, then individually glued onto the medical gauze within each of the two *Arabidopsis* quadrants. In each row of the quadrant, 12 equally spaced planting locations were planted with two seeds. There was no crew time allotted for thinning operations during the validation mission so only two seeds per location were planted.

#### Apogee Wheat Planting System

The planting and germination protocols for wheat were adopted and modified from those used in BPS during the PESTO experiment (Monje et al., 2005). Apogee wheat seeds (provided by Bruce Bugbee, Utah State University) were planted in two quadrants using CapMat II wicks (16.5 cm by 4 cm; Phytotronics, Inc., Earth City, MO, United States). The wheat seeds used were not sanitized because it was found that the ethanol wash protocol used for the *Arabidopsis* seeds decreased wheat seed germination down to 20%. Ten seeds were planted in each row for a total of 30 seeds in each quadrant. The wicks and Roylan foam were autoclaved using a 20 min dry cycle. Two CapMat strips were placed together inside slits cut in the Roylan foam to form two capillary wicks that held the seeds during launch and growth in the ISS. Each side of the seed was dipped in sterile 1% guar gum, and the seeds were planted with the embryo tip downward into the arcillite substrate, positioned so that the glued edges of the seeds were in contact with the CapMat II wicks. The wheat quadrants were located closest to the door of the APH growth chamber during the ISS flight experiment to allow plant height determination using the side-viewing IR camera.

#### Science Carrier Flight Configuration

Following seeding the SC was dried overnight in a sterile laminar flow bench to allow the glue holding the seeds to dry. Drying in the flow bench minimizes contamination of the planted SC with airborne contaminants. The SC was then sealed within a large, gas impermeable Tedlar bag (SKC, Inc., Eighty Four, PA, United States) (Figure 2E), and packed in foam for launch to the ISS.

## First Plant Test

### Background: Gas Exchange Measurements

In plant ecophysiology, gas exchange systems are used for measuring carbon and water vapor fluxes from photosynthetic organisms (Pearcy et al., 1989; Monje and Bugbee, 1998, 2019). These techniques have been used in the 1980s and 1990s during NASA’s Advanced Life Support Program to measure crop radiation use efficiencies and transpiration rates for estimating the size and feasibility of future BLSS (Averner et al., 1984; Rummel and Volk, 1987; Bugbee and Salisbury, 1988; Wheeler, 2010).

The APH was designed to permit the measurement of photosynthetic and transpiration rates from the plants within the GC. The system for controlling the CO_2_ concentration of the APH GC provides a means to measure canopy photosynthesis non-destructively using gas exchange techniques pioneered by ecophysiologists in the 1960s (Lange et al., 2001). Plants remove CO_2_ from the chamber atmosphere via photosynthesis when illuminated and add CO_2_ via respiration in the dark. The APH utilizes a semi-closed gas exchange system to maintain chamber CO_2_ setpoint, and acts like a closed system when CO_2_ control is disabled. In the semi-closed system, the chamber CO_2_ setpoint during the photoperiod is maintained by injecting small, known volumes of CO_2_ into the chamber and the photosynthetic rate is determined from the number of CO_2_ pulses injected over a given time period, but chamber leak rate must be known (Pearcy et al., 1989). At night, CO_2_ control is disabled and CO_2_ concentration rises due to dark respiration to a level higher than the daytime setpoint. At the start of the photoperiod when CO_2_ control and ethylene scrubbing are disabled, the chamber operates in closed mode and photosynthesis draws down the chamber CO_2_ concentration down to the daytime setpoint. This daily CO_2_ drawdown was used to measure photosynthetic rates during the development of the plant canopy growing in the APH.

### Spaceflight Growth Experiment

The primary goal of the first plant test was to verify that plants can be grown normally within the APH hardware on ISS. A principal investigator who has been awarded a spaceflight experiment utilizing the APH facility chooses the plant species, the environmental conditions for the life cycle, and supplies a pre-planted SC root module. The proposed science objectives are then demonstrated on the ground during science and experiment verification tests. The SC is then launched to ISS, inserted in the APH chamber by the crew, its water lines are connected to the WRADS, and temperature and soil moisture sensors from each quadrant are connected to the PHARMER. The crew closes the APH door and thereafter, the APH is configured by experiment profiles, which control the growth chamber environment and the root zone moisture level for the duration of the life cycle of the chosen plant species.

The first plant test was conducted in two stages using two plant species (Table 3). The *Arabidopsis* quadrants were watered first in order to verify the procedures for priming, germinating, growing *Arabidopsis* to maturity, and harvesting in microgravity in preparation for the PH-01 experiment. The *Arabidopsis* plants were grown for 17 days under low light (150 μmol m^–2^ s^–1^), low CO_2_ concentration (400 μmol mol^–1^) and a 16:8 photoperiod (Table 3), which are typical environmental setpoints for *Arabidopsis*. The germination rate of the *Arabidopsis* plant growth test was only 58%, which was significantly lower than the 80% germination rate observed in early germination tests on the ground. The germination rate reported is really a survival rate because germinating *Arabidopsis* plants are small and cannot be distinguished from background by the overhead camera. A greater number of plants may have actually germinated, but two reasons contributed to poor survival. Typically five plants are seeded per position, but only two were planted in this validation test to prevent overcrowding because crew time for thinning was not available. Poor survival was also observed due to uneven water distribution during priming of the SC and/or overwatering caused by microgravity-induced moisture redistribution phenomena during the early growth phases. These results were later addressed by the PH-01 research team during the implementation of their spaceflight experiment.

**TABLE 3:** Environmental setpoints and germination rates during the first plant test on APH.

Environment	*Arabidopsis*	Wheat	Units
Light intensity (PPFD)	150 ± 1.2	600 ± 1.1	μmol⋅m^–2 ⋅^s^–1^
Photoperiod (Light:Dark)	16:8	20:4	hr
CO_2_ concentration	400 ± 5.3	1500 ± 6.0	μmol mol^–1^
Temperature (Light:Dark)	22:18 (± 0.13)	23:23 (± 0.06)	°C
Humidity (%)	65 ± 0.9	75 ± 0.8	%
Experiment duration	48 (6 wk)	33	Days
Planting area	0.1	0.1	m^2^
# Plants grown/Planted	21/36	48/60	Plants
Germination rate	58	80	%

The wheat quadrants were imbibed when the *Arabidopsis* plants were 17 days old and grown at high light intensity (600 μmol m^–2^ s^–1^), elevated CO_2_ concentration (1500 μmol mol^–1^), and a 20:4 photoperiod (Table 3). Thus, *Arabidopsis* grew at high light, elevated CO_2_, and a longer photoperiod for ∼4 additional weeks, which are not normal conditions for this species. Furthermore, watering of the *Arabidopsis* quadrants was discontinued during the last week of *Arabidopsis* growth to minimize their contribution to gas exchange measurements made with wheat.

The *Arabidopsis* plants were harvested 6 weeks after planting, during flowering to demonstrate the ability of the APH ECS filter system to contain plant debris during harvest. Images revealed that plant debris was successfully trapped on the inlets to the ECS modules. These modules recirculate chamber air through an inlet protected by a screen and a HEPA filter. A video camera was placed outside the APH to detect debris flying out of the chamber; only one small flower was observed to leave the APH and it was captured by the crew. The inlets to the ECS modules were cleaned with a vacuum after the validation test was completed.

The germination rate of the wheat seeds was 80%, which was lower than the 92–97% germination rate observed in the BPS (Stutte et al., 2005). The wheat was grown until anthesis at 33 days after planting and grew next to the *Arabidopsis* plants for ∼25 days. The wheat plants imposed an increased water production load on the environmental control system (i.e., ECS) and an increased water demand load on the root zone watering system of APH (i.e., WRADS). The wheat canopy tested if the APH subsystems used for controlling chamber CO_2_ concentration and water fluxes were sized appropriately to maintain environmental setpoints when large plants are grown under microgravity conditions.

### Lighting System

Determining the light level regime within the growth chamber is important for interpreting photosynthetic measurements made in the APH because plant height changes during development. The GLA setpoints of the APH correspond to a light intensity measured 10 cm below the LED panel. However, the actual light intensity at plant height is determined by the degree of light attenuation versus height within the GC and by the height of the plants.

**FIGURE 3:**
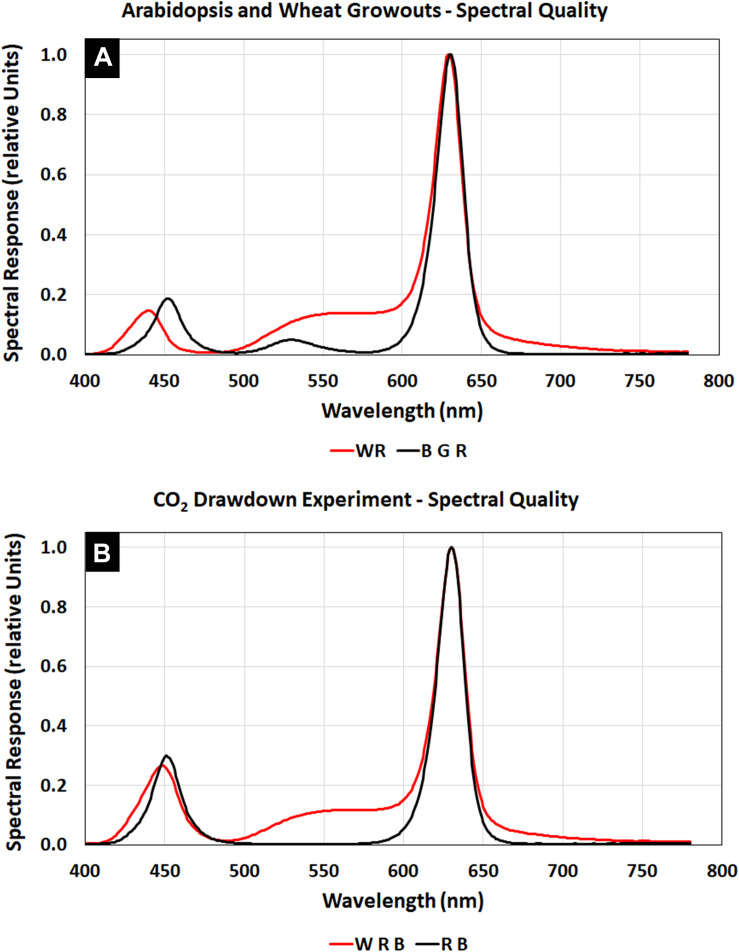
APH GLA spectra using White, Blue, Green, and Red LEDs: **(A)** Spectral quality during *Arabidopsis* (BGR, black line) and wheat (WR, red line) plant tests. **(B)** Spectral quality during the CO_2_ drawdown experiment (WRB, red line). Comparing the WRB and RB (black line) spectra shows that white LEDs supply additional blue and red light plus substantial amounts of green light.

The *Arabidopsis* plants were grown at a light level of 150 μmol m^–2^ s^–1^ with a spectral quality composed of blue, green, and red LED illumination (Figure 3A, BGR, black line), similar to that of Veggie (Figure 2I). The wheat/*Arabidopsis* canopy in the APH was grown at 600 μmol m^–2^ s^–1^ using a 1:1 ratio of white and red LEDs (Figure 3A, WR, red line). Two additional spectral compositions were used to study the light regime and to illustrate the spectral range provided by the APH GLA: (1) white LED supplemented with red and blue LEDs (Figure 3B, WRB red line), and (2) red and blue LEDs (Figure 3B, RB, black line). A comparison between the WRB and RB spectra shows that white LEDs supply additional blue and red light plus substantial amounts of green light, which penetrates deeper into plant canopies than red and blue light (Kim et al., 2006).

**FIGURE 4:**
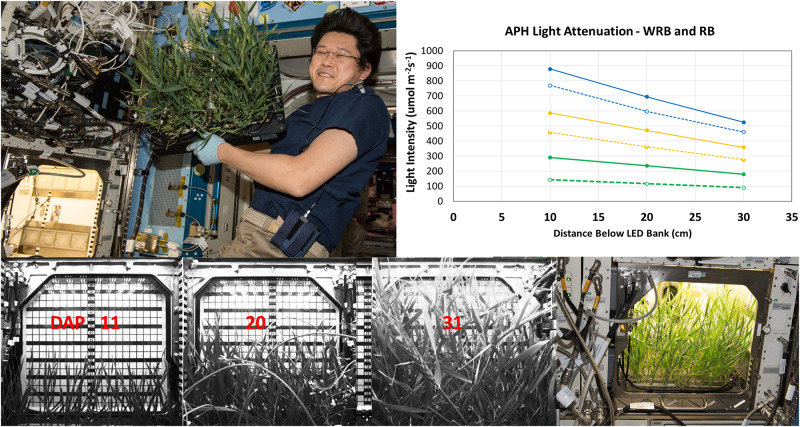
Light attenuation and plant height within APH. Light attenuation under WRB (solid lines) and RB (dashed lines), measured at 900 (blue), 600 (yellow), and 300 (green) μmol m^– 2^ s^– 1^ GLA setpoints, decreases linearly in APH. Wheat canopy height was measured using the grid of the APH door cover. Wheat was 15, 23, and 37 cm tall at 11, 20, and 31 DAP, respectively. The wheat plants were harvested after 33 DAP by Astronaut Norishige “Nemo” Kanai (Image Courtesy NASA used for informational purposes without explicit permission).

The APH ground unit was used to measure the degree of light attenuation for several GLA setpoints (900, 600, and 300 μmol m^–2^ s^–1^) of WRB (Figure 4, inset, solid lines) and RB (Figure 4, inset, dashed lines) illumination. These light attenuation curves show that light level below the GLA decreases in a linear fashion.

The plant height during development of the wheat canopy on ISS was estimated from images acquired using the side-facing IR camera. The height of the wheat canopy was measured using the grid placed on the inside of the APH door cover (Figure 4, bottom panel). The grid measures the height from the bottom of the GC to the GLA (45 cm), and the SC occupies 5 cm inside the GC, which leaves 40 cm between the top of the SC to the GLA. The plants were 15 cm tall (25 cm below the GLA) at 11 DAP; 23 cm tall (17 cm below GLA) at 20 DAP; wheat heads were observed at 37 cm (3 cm below GLA) on 31 DAP (Figure 4). For example, the plant heights at 11, 20, and 31 DAP correspond to upper canopy light levels of 337, 459, and 533 μmol m^–2^ s^–1^ at a GLA setting of 600 μmol m^–2^ s^–1^ under WRB illumination.

### CO_2_ Drawdown Experiments

#### Chamber Leak Rate

A CO_2_ drawdown experiment was performed following installation of the dry SC root module on the ISS to determine chamber leak rate of the APH GC. Chamber CO_2_ was raised to 2,000 μmol mol^–1^, CO_2_ control and ethylene scrubbing were disabled for 3 hr and chamber leak rate was measured according to Acock and Acock (1989). The leak rate of APH on ISS was 5% h^–1^, which is consistent with leak rates measured during ground studies. The leak rate must be known to correct changes in chamber CO_2_ during the CO_2_ drawdowns used to measure canopy photosynthesis.

#### CO_2_ Drawdown Technique

The CO_2_ drawdown technique is a non-destructive and non-invasive gas exchange technique for measuring photosynthetic rates from changes in chamber CO_2_ concentration. The CO_2_ control system operates the GC as a closed gas exchange system to determine: (1) daily canopy photosynthetic rates, and (2) response curves of photosynthesis to chamber CO_2_ concentration and to light intensity. During a CO_2_ drawdown curve, chamber CO_2_ is raised to 2,000 μmol mol^–1^, the plants are allowed to acclimate at the desired light level for ∼1 h, then chamber CO_2_ and ethylene control are disabled and a photosynthetic CO_2_ drawdown occurs. Ethylene control must be disabled because the permanganate-filled Purafil SP beads used to control ethylene absorb significant amounts of CO_2_ (Purafil, Inc., Doraville, GA, United States). Net photosynthetic carbon uptake consumes CO_2_ and chamber CO_2_ is reduced at a rate that is proportional to the incident light level. The change in CO_2_ concentration between 2,000 to 1500 μmol mol^–1^ is used to calculate canopy photosynthesis at a saturating CO_2_ concentration. For a CO_2_ drawdown experiment, repeating drawdown curves are conducted at descending light levels ending in a measurement of dark respiration.

#### Daily Canopy Carbon Fluxes in APH

**FIGURE 5:**
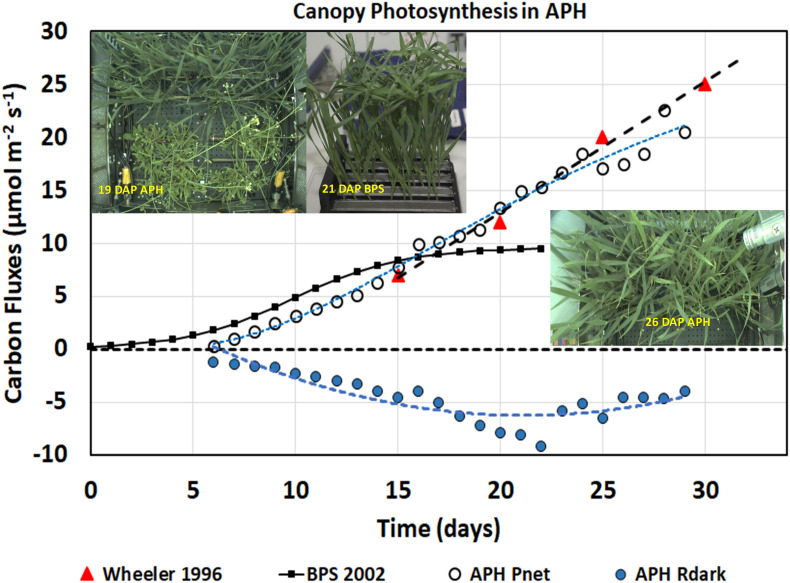
Daily photosynthesis and respiration measurements. Daily APH P_*net*_ measured from daily CO_2_ drawdown curves and dark respiration (R_*dark*_) rates increased with time as the wheat plants grew and fixed more CO_2_. The wheat/*Arabidopsis* canopy had similar gas exchange rates as wheat plants grown in spaceflight during BPS 2002 and on the ground by Wheeler, 1996.

After the 1st week of wheat growth, a single CO_2_ drawdown curve was programmed into the daily experiment profiles to measure daily rates of canopy photosynthesis at a saturating CO_2_ concentration of 1500 μmol mol^–1^. During the 4 h dark period, CO_2_ control was disabled for 2 h, allowing CO_2_ concentration to rise rapidly above 2,000 μmol mol^–1^. The initial rise in CO_2_ concentration vs. time during this period provided a measure of canopy dark respiration rate (Figure 5, APH R_*dark*_, blue circles). During the remaining 2 h of darkness, CO_2_ control was enabled at a setpoint of 1700 μmol mol^–1^. When the lights came on, CO_2_ control and ethylene scrubbing were disabled to initiate the drawdown curve. After 1 h, CO_2_ and ethylene control were enabled to re-establish a setpoint of 1500 μmol mol^–1^. The decrease in CO_2_ concentration vs. time during the drawdown period is a measure of maximal canopy net photosynthetic rate (Figure 5, APH P_*net*_, open circles). A 1 h limit for the daily CO_2_ drawdown was used because at high light the canopy can deplete CO_2_ rapidly, and it is imperative to prevent chamber CO_2_ concentration from dropping below the CO_2_ compensation point (∼100 μmol mol^–1^), which may cause photooxidative damage to the plants.

The daily canopy photosynthetic rates (Figure 5, APH P_*net*_, open circles) include CO_2_ fixation from 48 wheat and 14 *Arabidopsis* plants contained within the APH GC, each planted in 0.1 m^2^ (i.e., 50%) of the root module (Figure 5, inset 19 DAP APH). Donahue et al. (1997) reported a photosynthetic rate of 10–12 μmol m^–2^ s^–1^ for *Arabidopsis* grown at the same light intensity. Using these values, the *Arabidopsis* is estimated to have contributed between 40–58% of APH P_*net*_ observed during the first 15 DAP of wheat growth. The actual contribution from the *Arabidopsis* plants may have been less because they were senescing. After 20 DAP, the canopy photosynthesis was assumed to be primarily (∼80–90%) from the wheat plants, as watering of the two *Arabidopsis* SC quadrants had been discontinued and the *Arabidopsis* plants were beginning to desiccate. APH P_*net*_ after 26 DAP was entirely from wheat because the *Arabidopsis* plants were removed from the GC, however, dark respiration was probably influenced by root respiration from the desiccating *Arabidopsis* roots.

The daily canopy net photosynthetic rates measured in APH were compared to two wheat canopy photosynthetic experiments. The first is a wheat ground study conducted at a light intensity of 500 μmol m^–2^ s^–1^ using High Pressure Sodium (HPS) lamps (Figure 5; Wheeler 1996, red triangles). Wheeler (1996) reported photosynthetic rates of 7, 12, 20, and 25 μmol m^–2^ s^–1^ at 15, 20, 25, and 30 DAP, respectively. The second study is the 2002 PESTO spaceflight experiment conducted in the BPS using Cool White Fluorescent (CWF) lamps at a light intensity of 280 μmol m^–2^ s^–1^ (Figure 5, BPS 2002, black line) (Monje et al., 2005; Stutte et al., 2005).

The daily APH P_*net*_ rates from 7 to 15 DAP (Figure 5, white circles) were slightly lower than the rates measured in BPS (Figure 5, BPS 2002, black line), probably due to higher root respiration from the deeper and larger APH root modules. From 15 to 29 DAP, the daily P_*net*_ matches the photosynthetic rates of wheat grown at 500 μmol m^–2^ s^–1^ (Figure 5; Wheeler 1996, red triangles). After 15 DAP, daily P_*net*_ remains higher than was observed in the BPS. Photosynthesis in BPS was constant from 15 to 21 DAP because the wheat flag leaves were larger than the height of the BPS chamber, which caused the upper leaves to fold over each other resulting in self-shading (Figure 5, inset 21 DAP BPS), thus preventing higher penetration of light into the canopy. In contrast wheat grown in APH allowed more light to penetrate into the canopy (Figure 5, inset 26 DAP APH), thus P_*net*_ increases with age at a rate similar to that reported by Wheeler (1996). After 26 DAP, daily P_*net*_ was lower because the photosynthetic contribution from *Arabidopsis* was removed. These observations suggest that the wheat/*Arabidopsis* canopy grown during the first plant test of the APH had similar gas exchange rates as wheat plants grown on the ground.

#### APH CO_2_ Drawdown Experiment

A CO_2_ drawdown experiment was conducted to verify that the APH can measure responses of canopy photosynthesis to CO_2_ concentration and light intensity. The CO_2_ drawdown experiment was conducted when the wheat plants were 24 days old and the *Arabidopsis* plants were 41 days old at the daily settings of air temperature (23°C) and chamber humidity (75%). Two CO_2_ drawdown experiments were conducted on consecutive days to determine the repeatability of these measurements.

Each experiment consisted of a series of seven CO_2_ drawdown curves at decreasing light levels. The experiment profile changed the GLA setpoints for each LED (Table 4), as well as, enabled/disabled CO_2_ control and disabled ethylene scrubbing functions automatically. The plants were illuminated with WRB LEDs that provided a constant red:blue ratio of 5 (Figure 3B, WRB, red line). The incident radiation at canopy height (PPFD, Table 4) was held constant during each drawdown. The PPFD was estimated using the image analysis described in section “Lighting System” (Figure 4).

**TABLE 4:** Setpoints for the APH CO_2_ Drawdown Experiment^1^.

GLA Setpoint	White	Red	Blue	PPFD^2^
900	360	450	90	799
800	320	400	80	710
600	240	300	60	533
400	160	200	40	355
300	120	150	30	266
150	60	75	15	133
70	28	35	7	62

*^1^Units, μmol m^–2^ s^–1^. ^2^Light intensity at canopy height at 25 DAP.*

**FIGURE 6:**
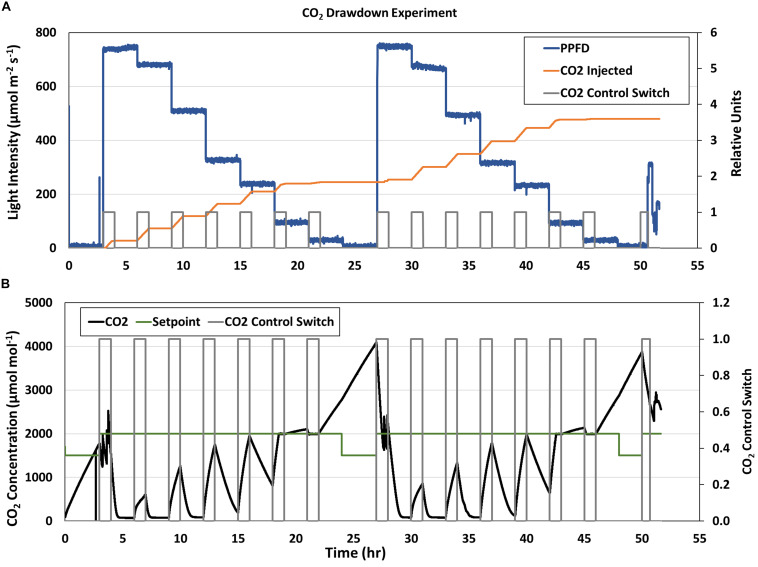
APH CO_2_ Drawdown Experiment. **(A)** Stepwise changes in GC light intensity (blue line) and **(B)** CO_2_ concentration (black line) during the two consecutive CO_2_ Drawdown experiments. The CO_2_ control switch [**(A,B)**, gray line] enabled CO_2_ injections [**(A)**, orange line] to raise chamber CO_2_ to 2,000 μmol mol^– 1^ before each drawdown. CO_2_ concentration decreased when CO_2_ control was disabled due to photosynthetic CO_2_ uptake. The CO_2_ injection rate during the second and third drawdowns was not sufficient to overcome canopy P_*net*_ at higher light levels and chamber CO_2_ concentration [**(B)**, black line] did not reach the CO_2_ setpoint [**(B)**, green line] in 1 h. As PPFD decreased, chamber CO_2_ reached the setpoint and even increased above it due to respiration.

The stepwise changes in PPFD during the CO_2_ drawdown experiments are shown in Figure 6A (blue line). The first drawdown curve at the highest GLA setpoint (900 μmol m^–2^ s^–1^) was conducted after the dark period from the previous day (4 and 28 hrs; Figure 6B). During subsequent drawdowns, the CO_2_ control system was enabled for 1 hr to raise chamber CO_2_ back to 2,000 μmol mol^–1^ (Figure 6A, gray box), and CO_2_ was indeed injected during those times (Figure 6A, orange line). However, the CO_2_ injection rate during the second and third drawdowns (at the 800 and 600 μmol m^–2^ s^–1^ light intensities) was not sufficient to overcome the rapid photosynthetic rates at the high light levels (7 and 10 hrs, Figure 6B, black line), and chamber CO_2_ concentration did not reach the CO_2_ setpoint (Figure 6B, green line) in 1 h. As PPFD decreased, chamber CO_2_ reached the setpoint and even increased above it due to respiration. The same trends were observed on the second day.

**FIGURE 7:**
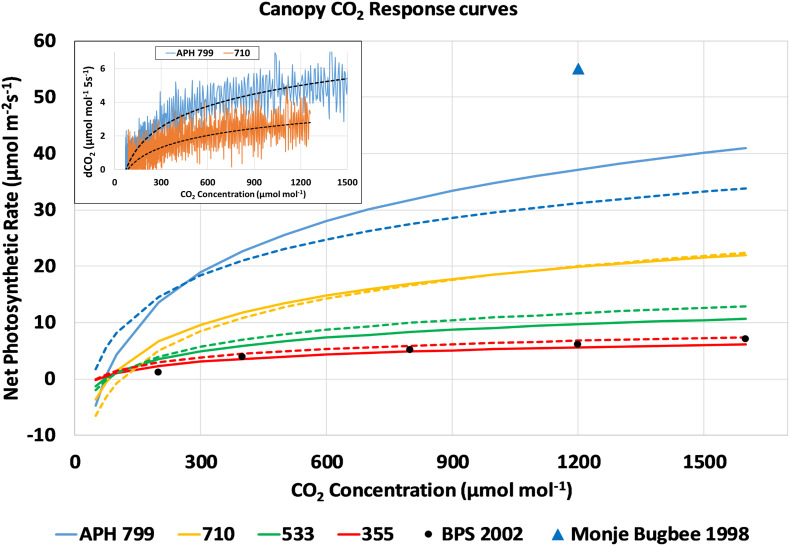
CO_2_ response curves of canopy photosynthesis during the CO_2_ drawdown experiment: Changes in chamber CO_2_ concentration (inset, dCO_2_ 5s^– 1^) from drawdown curves measured at constant PPFD were plotted vs. CO_2_ concentration, fitted with a logarithmic curve, and converted into photosynthetic CO_2_ response curves. Response curves measured during consecutive days are shown as solid and dashed lines. For comparison, the CO_2_ response curve measured during PESTO is plotted (BPS 2002, black circles) and P_*net*_ of wheat increases at higher light levels (Monje and Bugbee, 1998, blue triangle).

The drawdown curves obtained at each light level were used to calculate P_*net*_ from the change in chamber CO_2_ concentration during each 5 s interval (Figure 7, inset, dCO_2_ 5s^–1^). The dCO_2_ data was plotted vs. CO_2_ concentration, fitted with a logarithmic curve, and converted to a photosynthetic CO_2_ response curve assuming a 0.1 m^2^ planted area and a 100 L volume for the APH chamber. For simplicity, the approach used did not correct the dCO_2_ data for chamber leak rate, and it was assumed that the *Arabidopsis* plants did not contribute to the photosynthetic CO_2_ uptake. The resulting response curves of canopy photosynthesis to CO_2_ concentration for the four highest light levels are shown in Figure 7. The response curves measured during the first and second days are shown as solid and dashed lines, respectively. These CO_2_ response curves show that photosynthetic CO_2_ uptake is limited at lower CO_2_ concentrations, and that maximal rates of photosynthesis are affected by the incident radiation absorbed by the canopy (Figure 7). For comparison, the CO_2_ response curve measured during PESTO at 280 μmol m^–2^ s^–1^ PPFD (Figure 7, BPS 2002, black circles) is shown (Stutte et al., 2005). A single data point for the net photosynthetic rate of wheat measured at the same stage of development using HPS lamps at 1400 μmol m^–2^ s^–1^ PPFD and 1200 μmol mol^–1^ CO_2_ (Figure 7, blue triangle) shows that canopy photosynthesis can increase further at higher light levels (Monje and Bugbee, 1998).

**FIGURE 8:**
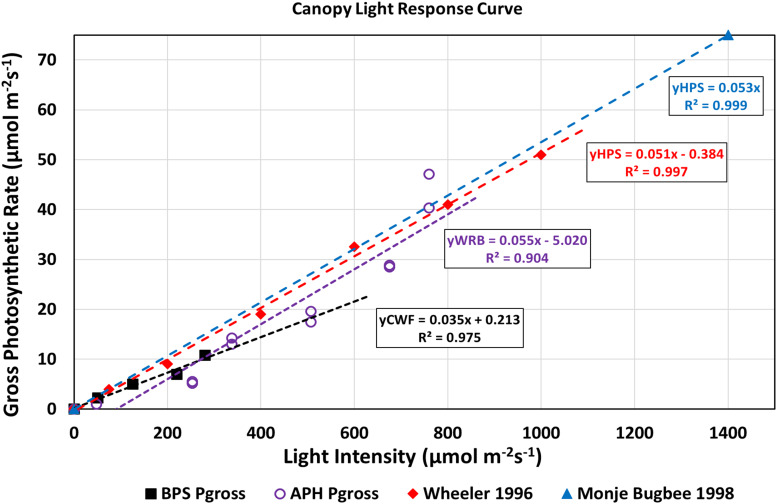
Light response curves of APH canopy photosynthesis: The APH CQY, the slope of gross photosynthesis vs. absorbed PPFD, represents the photosynthetic conversion of absorbed radiation into fixed CO_2_. The CQY of the wheat/*Arabidopsis* canopy measured in APH (WRB LEDs, purple open circles) is higher than reported in BPS 2002 (CWF lamps, black squares), but comparable to literature values of wheat CQY: Wheeler, 1996 (HPS lamps, red diamonds), and Monje and Bugbee, 1998 (HPS lamps, blue triangle).

Canopy light response curves describe how canopy photosynthetic rates, and therefore, crop growth rates vary as a function of incident radiation (Bugbee and Salisbury, 1988). They also provide information on the maximum photosynthetic capacity and CQY. A light response curve for the wheat/*Arabidopsis* canopy was constructed by plotting the gross photosynthetic CO_2_ uptake rate vs. absorbed radiation (Figure 8). Gross photosynthesis was calculated from the sum of P_*net*_ at 1500 μmol mol^–1^ CO_2_ (Figure 7) and the measured canopy dark respiration (R_*dark*_). The absorbed radiation was calculated assuming the wheat plants absorbed 95% of the incident radiation (Monje and Bugbee, 1998). The CQY, the slope of the plot of gross photosynthesis vs. absorbed PPFD, represents the photosynthetic conversion of absorbed radiation into fixed CO_2_. The CQY of the wheat/*Arabidopsis* canopy measured in APH (0.055; Figure 8, WRB LEDs, purple open circles) was compared to values of wheat CQY reported in the literature: Wheeler, 1996 (0.051; Figure 8, HPS lamps, red diamonds), BPS 2002 (0.035; Figure 8, CWF lamps, black squares), and Monje and Bugbee, 1998 (0.053; Figure 8, HPS lamps, blue triangle). The CQY values used in this comparison were determined from reported values of P_*net*_, R_*dark*_, and incident PPFD (Wheeler, 1996; Monje and Bugbee, 1998; Stutte et al., 2005).

The calculated APH CQY is higher than that measured in the BPS, probably because the wheat plants in the BPS were self-shaded. It is also slightly (4–8%) higher than the CQY of wheat grown under HPS lamps at 1g. The reason for these differences is that the gas exchange data collected includes error in the estimated incident radiation at the top of the canopy, error in the photosynthetic and respiration rates introduced by neglecting the leak rate correction, and errors from assuming that *Arabidopsis* did not contribute to the photosynthetic CO_2_ uptake measured in the APH. Another source of error in CQY is introduced from inclusion of the two partial drawdown curves because the photosynthetic rates obtained from those drawdown curves were not measured at the desired 1700 μmol mol^–1^ CO_2_ setpoints (Figure 6B). In spite of these shortcomings, the APH CQY measured in microgravity is in general agreement to CQY values obtained in wheat canopies grown on Earth at similar light intensities.

## Significance of the First Plant Test

The first plant test demonstrated the capabilities of the APH facility to conduct fundamental plant research in microgravity onboard the ISS. The hardware validation test verified that all subsystems of the APH were fully operational after it was assembled in the Kibo module. The first plant test demonstrated how two plant species requiring different optimum environmental conditions and with vastly different growth rates can be accommodated in the APH. Although there was minimal crew time for tending the plants, and no provisions for bringing plant samples for further analysis on the ground, the APH was able to measure plant responses to CO_2_ concentration and to light levels using non-destructive gas exchange techniques. The growth of the plants was accomplished via teleoperated commanding from the EMA at KSC, and the pre-programmed CO_2_ drawdown experiments were conducted using experiment profiles that controlled the setpoints required for measuring these responses. Admittedly, the gas exchange data collected during the first plant test was not repeated on the ground, thus it cannot be used to make any conclusions regarding plant growth in space. However, this effort demonstrated that the APH has expanded the capabilities of spaceflight plant growth chambers both in the size of the growth area, the higher light intensities and increased spectral combinations possible, as well as in their ability to collect non-destructive data sets (i.e., images and gas exchange rates) that can be used to measure plant growth during spaceflight. These capabilities will undoubtedly expand our knowledge of plant growth during spaceflight, which is needed for supporting the sustainable and long-term human colonization of space.

## Conclusion

The APH Facility was installed, assembled and its ability for conducting fundamental plant research on ISS was evaluated and demonstrated. Wheat and *Arabidopsis* plant canopies were successfully grown from seed and harvested after 6 weeks and after 33 days of growth on ISS, respectively. No pre-flight testing was performed on the ground, thus design team recommended settings were used throughout the operation of the APH during the validation test. The planting, germination, and watering protocols for the two species were demonstrated on ISS. The ability to grow *Arabidopsis* stems, flowers and siliques was accomplished in 6 weeks and the APH’s ability to contain debris during harvest was demonstrated during harvest of the mature *Arabidopsis* plants. Environmental and non-destructive plant growth data was collected and used to validate the ability of APH to measure photosynthesis, respiration and CQY of a plant canopy during spaceflight.

## Data Availability Statement

The datasets generated for this study are available on request to the corresponding author.

## Author Contributions

BO, ND, HL, JR, and OM contributed to the conception and design of the study. BO was the NASA APH Project Manager. ND was the NASA APH Project Engineer. HL was the NASA Project Scientist. OM and JR are members of the APH science team. JC was the Payload Integration Engineer and developed and managed the crew procedures. DD was the Jacobs Project Manager. OM analyzed the data, performed the statistical analysis, and wrote the first draft of the manuscript. JR, ND, HL, and BO wrote sections of the manuscript. All authors contributed to manuscript revision, read, and approved the submitted version.

## Conflict of Interest

The authors declare that the research was conducted in the absence of any commercial or financial relationships that could be construed as a potential conflict of interest.
